# Fusion Molecules of Heat Shock Protein HSPX with Other Antigens of *Mycobacterium tuberculosis* Show High Potential in Serodiagnosis of Tuberculosis

**DOI:** 10.1371/journal.pone.0163349

**Published:** 2016-09-21

**Authors:** Ruqyya Khalid, Madeeha Afzal, Sana Khurshid, Rehan Zafar Paracha, Imran H. Khan, Muhammad Waheed Akhtar

**Affiliations:** 1 School of Biological Sciences, Quaid-e-Azam Campus, University of the Punjab, Lahore, Pakistan; 2 Atta-ur-Rehman School of Applied Biosciences, National University of Sciences and Technology, Islamabad, Pakistan; 3 Department of Pathology and Laboratory Medicine, University of California, Davis, California, United States of America; University of Rochester, UNITED STATES

## Abstract

Variable individual response against the antigens of *Mycobacterium tuberculosis* necessitates detection of multiple antibodies for enhancing reliability of serodiagnosis of tuberculosis. Fusion molecules consisting of two or more antigens showing high sensitivity would be helpful in achieving this objective. Antigens of *M*. *tuberculosis* HSPX and PE35 were expressed in a soluble form whereas tnPstS1 and FbpC1 were expressed as inclusion bodies at 37°C. Heat shock protein HSPX when attached to the N-termini of the antigens PE35, tnPstS1 and FbpC1, all the fusion molecules were expressed at high levels in *E*. *coli* in a soluble form. ELISA analysis of the plasma samples of TB patients against HSPX-tnPstS1 showed 57.7% sensitivity which is nearly the same as the expected combined value obtained after deducting the number of plasma samples (32) containing the antibodies against both the individual antigens. Likewise, the 54.4% sensitivity of HSPX-PE35 was nearly the same as that expected from the combined values of the contributing antigens. Structural analysis of all the fusion molecules by CD spectroscopy showed that α-helical and β-sheet contents were found close to those obtained through molecular modeling. Molecular modeling studies of HSPX-tnPstS1 and HSPX-PE35 support the analytical results as most of the epitopes of the contributing antigens were found to be available for binding to the corresponding antibodies. Using these fusion molecules in combination with other antigenic molecules should reduce the number of antigenic proteins required for a more reliable and economical serodiagnosis of tuberculosis. Also, HSPX seems to have potential application in soluble expression of heterologous proteins in *E*. *coli*.

## Introduction

Tuberculosis (TB) represents an ongoing threat to global health, with the current epidemic fuelled by HIV-coinfection and an increasing incidence of drug-resistant strains of *M*. *tuberculosis*. In 2014, World Health Organization (WHO) reported 9.0 million cases of TB and 1.5 million deaths all over the world [1]. The standard of care for TB diagnosis recommended by WHO is (i) sputum smear microscopy for all cases, and (ii) expansion of the use of culture to diagnose all bacteriologically positive cases [2]. Microscopy has high specificity in TB-endemic countries, but modest sensitivity, which is quite variable among different laboratories. Also, the sensitivity is much lower among HIV-positive patients than among HIV-negative patients [3]. Culture is the most sensitive of currently available tests, but require long growth time and in 10–20% of cases the bacillus is not successfully cultured [4].

Serodiagnosis could offer solutions to some of these problems. Screening tests to overcome diagnostic delay, specific tests for diagnosis of extrapulmonary TB and other bacteriologically negative cases, and tests for vaccine-induced immunity need critical consideration [5]. Antibody detection based tests are potential diagnostic tools for tuberculosis but they lack sensitivity and specificity because of changes in antibody response to the same antigen in different individuals and at different stages of the disease [5, 6]. With the complete genome sequencing of the *M*. *tuberculosis* H37Rv strain, considerable progress has been made in the identification and evaluation of serological antigens. It is repeatedly observed that more than one antigen should be included in the ELISA-based serodiagnosis of tuberculosis. Therefore, the fusion protein molecule comprising of regions from two or more antigens may be helpful in increasing the sensitivity of diagnostic assays [7, 8]. Due to the inconsistent and variable results of ELISA kits, WHO recommended that these tests should not be used for diagnosis of TB. However, they stated clearly in their 2011 policy that further research to identify new/alternative point-of-care tests for TB diagnosis and/or serological tests with improved accuracy is strongly encouraged [2].

Several recombinant antigens have been identified that have diagnostic and prophylactic utility. Due to pathogenic nature of *M*. *tuberculosis*, recombinant production of serodiagnostic antigens in *E*. *coli* is a safe method; however, there are limitations due to low expression levels and expression of some of these as insoluble aggregates. Many important membrane associated serodiagnostic antigens of *M*. *tuberculosis*, having large hydrophobic areas, are expressed in the insoluble form in *E*. *coli* [9, 10]. To obtain good sensitivity of the assays, it is necessary that the antigens must be pure and in correctly folded form. Utilization of highly soluble protein as a fusion partner with insoluble protein had been explored for improving solubility, easy purification and enhancing immunogenicity. Many proteins like GST, *E*. *coli* trigger factor (TF), heat shock proteins or molecular chaperones have been fused to the protein of interest to get soluble and high level expression in *E*. *coli* [11–13]. Additionally, it is essential that the protein being fused to the antigens should not add any undesired immunodominance leading to false positive results.

Heat shock protein X (HSPX) belongs to the HSP20 family, also referred to as alpha crystallin protein family, and is the first member of this family to be identified in *M tuberculosis*, therefore, it is often referred to as alpha-crystallin 1 (acr1). Immunodominant HSPX antigen is a cytosolic protein and has chaperone-like activity [14, 15]. Serodiagnostic potential of HSPX has been widely studied [14, 16]. The sensitivity of HSPX for detection of IgG antibodies in pulmonary TB patients has also been reported to be variable, ranging from 34% [17] to 62% [18]. Together with other antigens, HSPX has been used to develop commercial serodiagnostic tests e.g., Pathozyme TB complex. B-cell epitopes from HSPX have been identified through peptide microarray technique [19, 20]. PE35 is present in the RD1 region of *M*. *tuberculosis* genome and has shown good sensitivity in detecting antibodies in plasma samples of TB patients as compared to BCG- vaccinated healthy controls [21]. FbpC1 can detect antibodies in plasma samples of advanced TB stages including HIV co-infection [22]. PstS1 is one of the earliest known immunodominant antigens [23, 24]. It is a lipoprotein antigen [25], specific only to the cavitary TB patients [26, 27]. We had shown previously that truncated or tnPstS1 had higher sensitivity in detecting antibodies in plasma samples of TB patients [28].

In this study, we expressed the individual HSPX, PE35, tnPstS1 and FbpC1 antigens as well as three novel fusion molecules i.e. HSPX-PE35, HSPX-tnPstS1 and HSPX-FpbC1 and evaluated these for their potential in detecting antibodies in plasma samples from TB patients.

## Materials and Methods

Ethical approval for this work was obtained from Ethical Review Committee, School of Biological Sciences, University of the Punjab Lahore, Pakistan, approval letter number SBS/987/11. Written informed consent was taken from all the study participants.

### Design and cloning of individual and fusion antigenic proteins

Full-length *HSPX* (435bp), *PE35* (300bp) and FbpC1 (818bp) were PCR amplified using their respective primers as described in Table 1.

**Table 1:** Primers used in PCR with the restriction sites shown as underlined.

Primers		Sequence	Restriction sites	Annealing temperature
**HSPX**	F1	GAGGCATCATATGGCCACCAC	*Nde*I	58
	R1	ACGGACCCAGTGG*TCA*GTTGG	None	62
	R2	GTAGGATCCGTTGGTGGACCGGAT	*Bam*HI	57
**PE35**	F2	CATATGGAAAAAATGTCACATGATCCG	*Nde*I	64
	F3	GGTGGATCCATGGAAAAAATGTCACA	*Bam*HI	64
	R3	GTGATCACTCCCTCCGATGTGTTGG	None	69
	R4	AACAAGCTTGATCACTCCCTCCGATGT	*Hind*III	68
**tnPstS1**^a^	F4	CATATGAAAATTCGTTTGCATACGCTGTTGG	*Nde*I	68
	F5	GTAGGATCCGCCGGGACGGTCAACATTG	*Bam*HI	61
	R5	TATAAGCTTCTACGCGGGCGGCAGCGGCTG	*Hind*III	68
**FbpC1**^b^	F6	TAACATATGGCCCCATACGAGAACCTG	*Nde*I	68
	F7	TAAGGATCCGCCCCATACGAGAACCTG	*Bam*HI	71
	R6	GCTCGCATCGGCACCTGGCTTAG	None	65
	R7	CACCAAGCTTAGCGGATCGCACCGACG	*Hind*III	74

As reported previously

^a^[28],

^b^[29]

Purified DNA fragments corresponding to *HSPX*, *PE35* and *FpbC1* were first cloned into pTZ57R/T cloning vector and then sub-cloned into pET28a(+) expression vector. Cloning of 792bp fragment of *PstS1* (composed of truncated sequence of PstS1 containing its major epitopes) into pET28a(+) was done as described previously [28].

**Fig 1:**
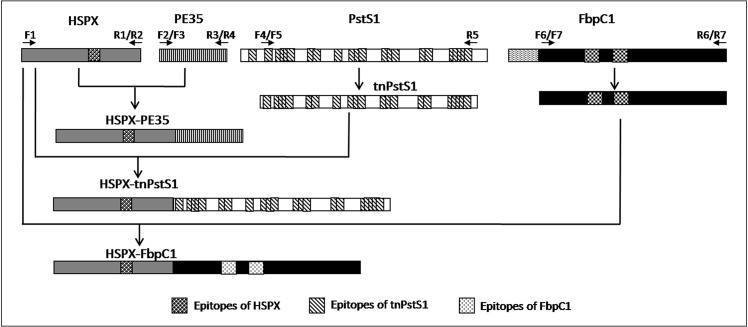
Scheme for the construction of the fusion molecules HSPX-PE35, HSPX-tnPstS1 and HSPX-FbpC1.

For the construction of fusion molecules, the gene encoding *HSPX* was amplified using F1 and R2 primers. The reverse primer lacked stop codon. *PE35* was amplified using F3 and R4 primers. *tnPstS1* was PCR amplified using F5 and R5 primers and *FbpC1* was amplified using F7 and R7 primers (Table 1). The Purified DNA fragment encoding HSPX was restricted with *Nde*I and *Bam*HI and ligated with linearized pET28a(+) resulting in the pHSPX vector. *PE35*, *tnPstS1*and *FbpC1*were restricted with *BamH*I and *Hind*III and ligated into linearized pHSPX resulting in pHSPX-PE35, pHSPX-tnPstS1 and pHSPX-FbpC1, respectively (Fig 1). The sequence and correct orientation of insert DNA were confirmed through sequencing using Beckman Coulter CEQ8000^™^ Genetic analyzer [30].

### Expression and purification of recombinant antigenic proteins

*E*. *coli* BL21 codonplus (RIPL) cells were transformed with the recombinant plasmids and grown in LB broth supplemented with kanamycin (50 μg/μl). The cells in the shake flask culture were induced with 0.5 mM IPTG, when OD_600_ reached ~0.6, and grown further at 37°C for 4–6 hours. Expression was also studied by growing the culture at low temperatures of 20°C and 25°C for 18 hours.

The harvested cells were washed with wash buffer containing 20 mM Tris-Cl, 0.5 M NaCl (pH 8.0) and then lysed ultrasonically in the same buffer containing 5 mM imidazole [31]. After cell lysis, the supernatant was collected for soluble HSPX, PE35, FbpC1, HSPX-PE35, HSPX-tnPstS1 and HSPX-FbpC1 antigens and purified through nickel chelating chromatography followed by anion exchange FPLC. Insoluble tnPstS1 was purified after solubilization and refolding, as described previously [28]. Total cell protein, soluble and insoluble protein fractions and purified protein samples were analyzed on SDS-PAGE. The protein concentration was estimated by Bradford assay using bovine serum albumin as standard [32]. The percentage of the expressed protein was determined by scanning the total cell proteins on SDS-PAGE using Syngene gel documentation system (United Kingdom).

### Reactivity with anti-HSPX, anti-PstS1 and anti-FbpC1 polyclonal antisera

Antibodies against the native antigens HSPX, PE35, PstS1 and FbpC1 antigens were raised in rabbits, as described previously [28]. Reactivity of the individual and the fusion antigenic proteins with rabbit anti-HSPX, anti-PstS1 and anti-FbpC1 polyclonal antisera was checked through ELISA and western blotting [28].

### CD spectroscopy

The CD spectra of the individual and the fusion proteins were taken using the ChiraScan Plus CD Spectrophotometer (Applied Biophysics, UK). The final concentrations of protein samples of HSPX, tnPstS1, FbpC1, HSPX-PE35 and HSPX-FbpC1 were 0.4 mg/ml. For PE35 and HSPX-tnPstS1, the final concentrations were 0.2 mg/ml in 10 mM Tris-Cl (pH 8.0). A full scan was taken over the wavelength range 190–280 nm at 20°C, using the quartz cell of 1 mm path length. Each wavelength spectrum was the result of averaging two consecutive scans with a bandwidth of 1 nm. The wavelength spectra were refined by subtracting the blank spectra obtained with the buffer only. The secondary structure was then calculated using CDNN [33] which calculates secondary structures by comparing with the CD database of the known protein structures.

### Detection of antibodies in human plasma

The plasma samples of 180 patients diagnosed with pulmonary TB based on the signs and symptoms and confirmed through culturing of *M*. *tuberculosis* on LJ medium were collected from Gulab Devi Hospital, Lahore, Pakistan. All the samples were collected at the start of treatment for tuberculosis. 100 BCG-vaccinated healthy individuals, who had no prior history of close contact with TB patients and had no clinical history, were included in the control group. Informed consent was taken from all the study participants. ELISA was performed with plasma samples of TB patients, as described previously [28] except that dilution of HRP conjugated anti-human IgG secondary antibody was 1:5000 and the reaction with TMB was stopped after 7 minutes.

### Data analysis

For calculating the cutoff values, optical density (OD_450/630_) values from healthy controls were analyzed to calculate mean and standard deviation. The cutoff value for positive samples was the mean plus 2.576 multiples of the standard deviation. All the values were normalized by dividing each with that of the cut off. A sample showing value greater than one was considered positive. Specificity was calculated by dividing the number of controls found negative by the total number of healthy controls [14, 29, 34]. The results were also analyzed by plotting the Receiver Operating Characteristic (ROC) curve using GraphPad Prism 6 for windows (Graph-Pad software Inc., San Diego, CA). Comparison between the antibody titer of both groups was measured by non-parametric Mann-Whitney test (SPSS version 17).

### In silico structural analysis

For the homology modeling of the fusion proteins HSPX-PE35, HSPX-tnPstS1 and HSPX-FbpC1, crystal structures of the proteins homologous to the constituent proteins were searched from the protein data bank by using Protein Specific Iteration Blast (PSI-BLAST). Crystal structure of heat shock proteins from *Schizosaccharomyces pombe* (PDB ID: 3W1Z) [35] and another protein of eukaryotic origin (PDB ID: 1GME) [36] found homologous to the HSPX region which were used in its modeling. As homologous crystal structure of PE35 protein could not be found, the model for this protein was build using the Robetta server (http://robetta.bakerlab.org/) [37]. ABC phosphate transport receptor (PDB ID: 1PC3) [38] of *M*. *tuberculosis* whose crystal structure is known and also has 100% identity with tnpstS1 was used in the modeling of HSPX-tnPstS1 fusion protein. Crystal structure of FbpC1 (PDB ID: IR88) was used in the modeling of HSPX-FbpC1 fusion protein [39].

To evaluate the possible domain-domain interactions of fusion proteins, we employed the same strategy as used previously [40]. Firstly, the protein binding sites were determined using CPORT [41]. HADDOCK [42] web server was used to perform protein-protein interactions, which produced complexes. Based on the scores of HADDOCK, best complexes were selected and used in the modeling of full-length fusion proteins by MODELLER [43]. Overall, 25 models were generated and clustered using NMRCLUST [44]. Final selection of the models for each fusion protein was based on the scores provided by MODELLER, RAMACHANDRAN PLOT, ERRAT [45] and QMEAN [46]. CPORT (http://haddock.science.uu.nl/services/CPORT) [41] and ASAView (http://www.abren.net/asaview/) [47] were used to evaluate solvent accessible surfaces of fusion proteins.

## Results

### Expression and purification

**Fig 2:**
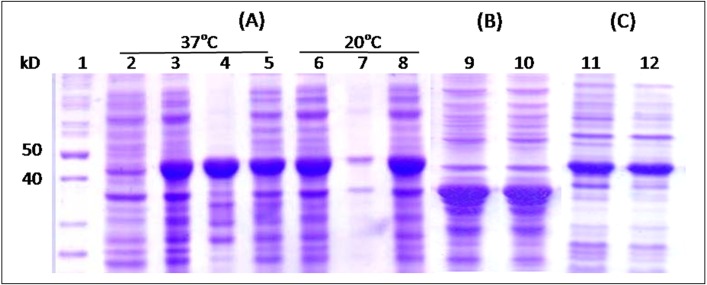
SDS PAGE analysis. Total, soluble and insoluble proteins of *E*. *coli* cells expressing His-HSPX-tnPstS1 (A), His-HSPX-PE35 (B) and His-HSPX-fbpC1 (C). 1- Protein size markers; 2- Uninduced cells; 3, 6, 9, 11- total cell proteins; 4, 7- insoluble fraction after cell lysis; 5, 8, 10, 12- soluble fraction of the cell lysate.

SDS-PAGE analysis of the total proteins of *E*. *coli* cells after transformation showed expression levels for His-HSPX, His-PE35, His-tnPstS1 and His-FbpC1 at 44%, 23%, 25% and 39%, respectively (Table 2). His-HSPX and His-PE35 were expressed in soluble form whereas His-tnPstS1 was expressed in insoluble form at 37°C as well as at 18°C as reported previously [28]. His-FbpC1 was expressed in insoluble format 37°C, but at 25°C it was expressed in a soluble form [29]. His-HSPX-PE35, His-HSPX-tnPstS1 and His-HSPX-FbpC1were expressed at the levels of 34%, 38% and 36% of the total cell proteins, respectively. All the fusion molecules were produced in a soluble form (Fig 2).

**Table 2:** Expression and purification of native antigens of *M*. *tuberculosis* and their fusion proteins.

Purification steps	His-HSPX	His-PE35	His-tnPstS1^a^	His-FbpC1^b^	His-HSPX-PE35	His-HSPX-tnPstS1	His-HSPX-FbpC1
**Expression level (%)**	44	23	25	39	34	38	36
**Yield (mg/L/OD**_**600**_**)**	79.2	41.4	45	70.2	61.2	68.4	64.8
**Purity (%)**	>90	>80	>90	>90	>80	>90	>90
**Recovery (%)**	39.5	24	38.4	25	37	30	35
**Amount of purified protein (mg/L/OD**_**600**_**)**	31.20	9.84	17.28	17.5	22.5	20.52	22.68

As reported previously

^a^[28],

^b^[29]

After purification through nickel chelating chromatography followed by anion exchange FPLC, all the proteins were >90% purified except PE35 and HSPX-PE35 which were >80% purified as shown in Table 2.

Amounts of the expressed HSPX, PE35, tnPstS1, FbpC1, HSPX-PE35, HSPX-tnPstS1 and HSPX-FbpC1in *E*. *coli* were 79.2, 41.4, 45, 70.2, 61.2, 68.4 and 64.8 mg/L/OD_600_, respectively. However, the net recoveries after purification were 31.2, 9.84, 17.28, 17.5, 22.5, 18.83 and 22.68 mg/L/OD_600_ for HSPX, PE35, tnPstS1, FbpC1, HSPX-PE35, HSPX-tnPstS1 and HSPX-FbpC1, respectively (Table 2).

### Reactivity with rabbit polyclonal antisera

**Fig 3:**
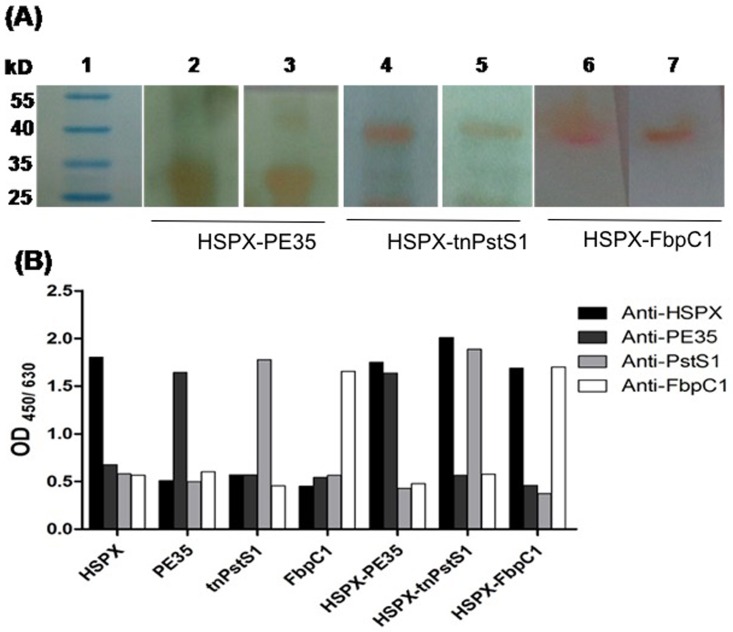
Antibody response of individual and fusion proteins with polyclonal antisera. (A) Western blot analyses of HSPX-PE35, HSPX-tnPstS1 and HSPX-FbpC1 with anti-HSPX (lanes 2, 4 & 6); anti-PE35 (lane 3); anti-tnPstS1 (lane 5) and anti-FbpC1 (lane 7). (B) Bar graph of OD_450/630_ obtained from ELISA of individual and fusion antigens against anti-HSPX, anti-PE35, anti-PstS1 and anti-FbpC1.

HSPX-PE35 fusion protein reacted equally well with both anti-HSPX and anti-PE35 polyclonal antibodies raised in rabbits as determined by western blotting and ELISA (Fig 3). Likewise, HSPX-tnPstS1 fusion protein reacted with both anti-HSPX and anti-PstS1 polyclonal antibodies (Fig 3). Similarly, HSPX-FbpC1 fusion protein reacted with both anti-HSPX and anti-FbpC1 polyclonal antibodies (Fig 3).

### CD spectrum of antigen variants

**Fig 4:**
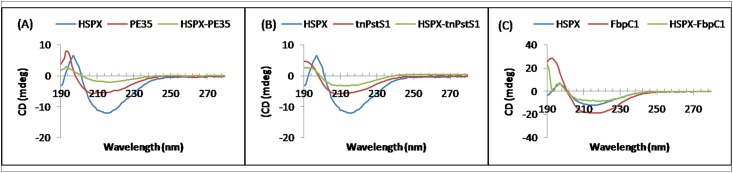
Circular dichroism spectra of individual and fusion proteins. CD spectrum of HSPX, PE35 and HSPX-PE35 (A); HSPX, tnPstS1 and HSPX-tnPstS1 (B) and HSPX, FbpC1 and HSPX-FbpC1 (C) over the range of 190nm to 280nm.

The CD spectra of all the individual proteins and their fusion molecules are given in Fig 4. Table 3 shows the percentages of α- helices and β- sheets of all the protein variants predicted through CDNN software and their molecular models or known structures.

**Table 3:** Percentages of secondary structures analyzed through CD spectrum and from predicted molecular models.

Fusion antigens	CD spectrum	Molecular modeling
**HSPX-PE35**	30.3% α- helices 23.5% β- sheets	36.9% α- helices 16.6% β- sheets
**HSPX-tnPstS1**	23.5% α- helices 21.6% β- sheets	25.1% α- helices 23.7% β- sheets
**HSPX-FbpC1**	23.7% α- helices 20.1% β- sheets	25.9% α- helices 17.3% β- sheets

### Analysis of plasma samples of TB patients

**Fig 5:**
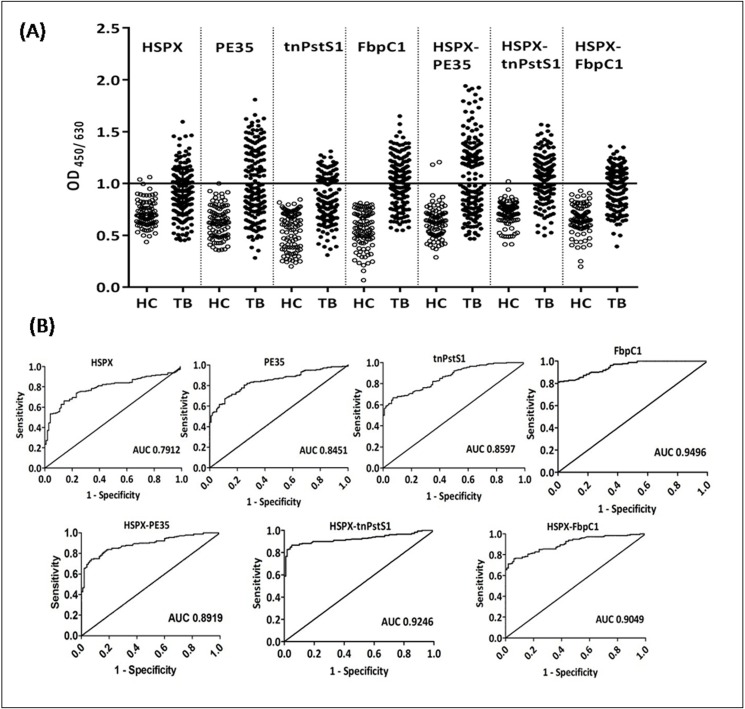
Analysis of antibody response of individual and fusion proteins in human plasma. Analysis of antibody response of the individual HSPX, PE35, tnPstS1 and FbpC1; and their fusion molecules HSPX-PE35, HSPX-tnPstS1 and HSPX-FbpC1 in human plasma. (A) Scatter dot plot of values of normalized OD_450/630_ of healthy controls (HC) and TB patients with individual and fusion proteins. (B) ROC curve of individual and fusion proteins.

The antibody response to all the individual and fusion antigens was significantly high (P < 0.0001) for plasma samples of TB patients as compared to those of healthy controls. Out of 180 plasma samples of TB patients 56, 82, 76 and 108 were positive for HSPX, PE35, tnPstS1, and FbpC1, respectively. Thus, 31.1, 45.5, 42.2 and 60% sensitivities were found for HSPX, PE35, tnPstS1 and FbpC1, respectively. In the case of fusion proteinsHSPX-PE35, HSPX-tnPstS1 and HSPX-FbpC1, 98, 103 and 100 were found positive with corresponding sensitivities of 54.4%, 57.2% and 55.5%, respectively (Fig 5A and Table 4).

**Table 4:** Sensitivity and specificity of the individual and the fusion antigens in detecting tuberculosis.

Antigen variants	Sensitivity %			Specificity %
	Total TB samples n = 180	Smear positive n = 94	Smear negative n = 86	
**HSPX**	56 (31.1%)	33 (35.1%)	23 (26.7%)	98
**PE35**	82 (45.5%)	48 (51.0%)	34 (39.5%)	100
**tnPstS1**^a^	76 (42.2%)	45 (47.8%)	31 (36.0%)	100
**FbpC1**^b^	108 (60%)	67 (71.2%)	41 (47.6%)	100
**HSPX-tnPstS1**	104 (57.7%)	61 (64.8%)	43 (50.0%)	99
**HSPX-PE35**	98 (54.4%)	56 (59.5%)	44 (51.1%)	98
**HSPX-FbpC1**	100 (55.5%)	52 (55.3%)	48 (55.8%)	100
HSPX+tnPstS1*	100 (55.5%)	58 (61.7%)	41 (47.6%)	99
HSPX+PE35*	94 (52.2%)	55 (58.5%)	41 (47.6%)	98
HSPX+FbpC1*	130 (72.22%)	79 (84.04%)	51 (59.30%)	98

*Combined sensitivities were corrected by reducing the number of the samples which showed presence of both antibodies. As reported previously

^a^[28],

^b^[29].

ROC curve for individual proteins showed the values of 0.7892, 0.8010, 0.8594 and 0.9694 for HSPX, PE35, tnPstS1 and FbpC1, while the ROC value for fusion proteins HSPX-PE35, HSPX-tnPstS1 and HSPX-FbpC1 were 0.8919, 0.9246 and 0.9049, respectively (Fig 4B).The values of healthy controls obtained higher than the cutoff were 2 for HSPX but none in the case of PE35, tnPstS1 and FbpC1. In the cases of HSPX-PE35, HSPX-tnPstS1and HSPX-FbpC1, only 2, 1and none of the healthy controls, respectively, showed values higher than the cutoff. Specificities, therefore, ranged between 98–100% in all the cases (Fig 5A and Table 4).

### Molecular models of fusion proteins

**Fig 6:**
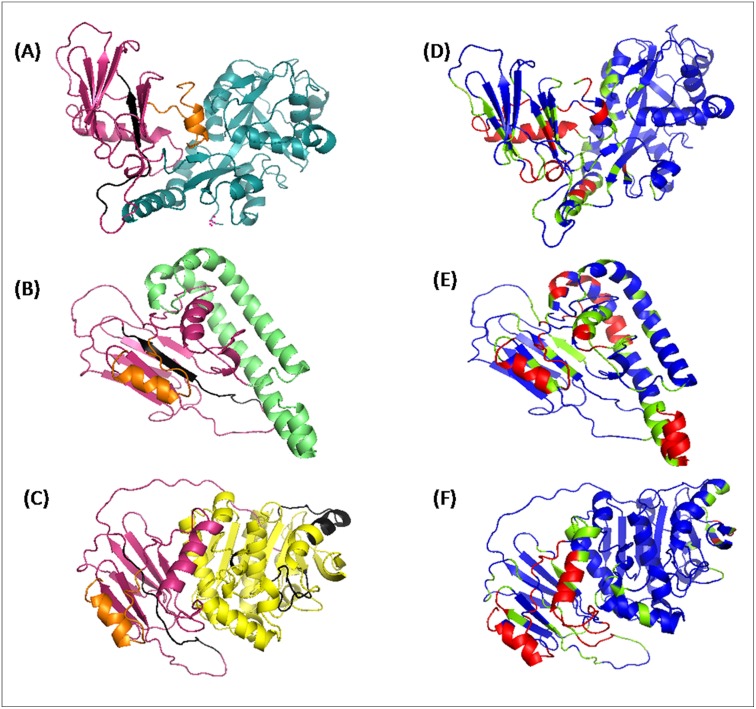
Molecular model of fusion proteins and their CPORT analyses. (A-C) Molecular models of HSPx-tnPstS1 (A), HSPX-PE35 (B) and HSPX-FbpC1 (C). HSPX in each of the three fusion protein is shown as pink, whereas tnPstS1, PE35 and FbpC1 are shown in cyan, green and yellow color respectively. CPORT analyses of these fusion molecules (D-F) show the region marked as red, green and blue that are active, supporting and non-supporting, respectively in antibody binding. The epitope region in HSPX-tnPstS1 (D) and HSPX-PE35 (F) are shown mostly in red or green. However, the epitope regions of HSPX-FbpC1 Shown in black (C) appear blue in F.

Based on the scores provided by MODELLER, RAMACHANDRAN PLOT, ERRAT, 25 out of 100 models were selected for further refinement in terms of energy minimization and protonation of structures. Further structural refinement resulted in the models of fusion molecules which are shown in Fig 6A–6C. The Solvent accessibility of the fusion proteins as observed through CPORT is given in Fig 6D–6F. Red colored residues are actively involved in any type of interaction; green colored residues support the interaction while blue colored residues are non-supporting. It was found that the epitopes of contributing proteins in HSPX-tnPstS1 and HSPX-PE35 are accessible (as most of the epitope region in red or green color) and thus indicating their active involvement in antigen-antibody interactions. In the case of HSPX-FbpC1, the epitopes of FbpC1seem to be relatively less accessible, as most of the epitope region was shown as blue.

## Discussion

For achieving the high level of reliability for serodiagnosis of tuberculosis multiple antigens of *M*. *tuberculosis* must be used. This is due to the variable immune response among different individuals. Therefore, fusion molecules constructed from two or more antigens of *M*. *tuberculosis*, with the epitopes of all the contributed antigens fully accessible to the corresponding antibodies, should make serodiagnosis more reliable. We expressed three *M*. *tuberculosis* antigens PE35, tnPstS1 and FbpC1 in fusion with heat shock protein, HSPX. Expression of the fusion molecules, HSPX-PE35 and HSPX-tnPstS1, was increased by about 50% as compared to those of the individual molecules PE35 and tnPstS1 (Table 2). This would be a significant contribution toward the cheaper production of the fusion antigens. Like PE35, His-HSPX-PE35 was expressed soluble at 37°C. His-HSPX-FbpC1 was also expressed soluble at 37°C, although the native FbpC1 was expressed soluble at 25°C requiring a longer fermentation period [29]. Strikingly, tnPstS1 in fusion with HSPX was expressed partially soluble form at 37°C, while it was expressed almost totally soluble form by lowering the growth temperature to 20°C. PstS1 was previously reported to be expressed in the soluble form using *E*. *coli* TF as fusion partner [12]. However, use of TF, not being an *M*. *tuberculosis* antigen, could lead to undesired immunodominance and false positive results. Fusion proteins involving HSPX, being an *M*. *tuberculosis* antigen, would be a more favorable choice. Soluble expression of fusion proteins is likely to be due to chaperone activity of the heat shock protein assisting in the conformational processing of newly synthesized protein by preventing non-productive hydrophobic interactions and help to gain its correct tertiary conformation [48].The fusion of PE35 and tnPstS1 with HSPX resulted in higher expression and recoveries of the fusion proteins as shown in Table 2. Use of the fusion molecules should, therefore, make the serodiagnostic procedure more reliable and economical.

The reactivity of fusion antigens with their respective antisera suggests that the epitopes of the contributing antigens appear to be functional in all the fusion molecules. Analysis of all the 180 plasma samples of TB patients showed that 56 samples (31.1%) were positive for HSPX and 76 (42%) for tnPstS1. Out of these 132 positive samples, 32 contained the antibodies for both the antigens. Therefore, the combined sensitivity for the two antigens shall be 55.5% calculated on the basis of 100 positive samples out of 180. The sensitivity value for HSPX-tnPstS1 determined experimentally was 57.7%, which is close to expected combined sensitivity of the two individual antigens (55.5%) as shown in Table 4. Similarly, the combined number of positive plasma samples containing antibodies for HSPX or PE35 after deducting the number of samples positive for both of the antigens was quite similar to the total number of samples detected with fusion protein HSPX-PE35 (~54%) as shown in Table 4. This data suggests that most of the epitopes of the two contributing proteins in both the fusion molecules, HSPX-tnPstS1 and HSPX-PE35, are available for binding to the corresponding antibodies.

However, in the case of HSPX-FbpC1, out of 180 samples, 56 were positive for HSPX and 108 were positive for FbpC1. Out of these positive samples, 34 were found positive for both antigens. Therefore, expected combined sensitivity for these two antigens is 72.2% which is higher than that of the fusion molecule HSPX-FbpC1 (55.5%). A lower sensitivity of this fusion protein as compared to that of FbpC1 indicates masking of the epitopes resulting from the specific arrangement of the two proteins against each other. Orientation of the fusion molecule on binding to the ELISA plate as well as the antigen aggregation may affect the presentation of epitopes to the antibody molecules and contribute to a lower sensitivity of fusion molecule as compared to those of its native counterparts [49].

AUC value obtained from ROC curve illustrates the efficiency of the diagnostic test which should make a clear distinction between the samples with or without disease. The AUC value of 1 represents a perfect test and of 0.5 represents a useless test. ROC curve of fusion protein HSPX-tnPstS1 and HSPX-PE35 showed higher AUC value of 0.9246 and 0.8919 as compared to those of their contributing individual proteins as shown in Fig 5B. The higher AUC value of fusion proteins implies that these can detect TB patients more accurately as compared to those of individual proteins.

Further conformational analyses of the three fusion proteins, HSPX-PE35, HSPX-tnPstS1 and HSPX-FbpC1, through molecular modeling showed that both the contributing proteins in each of these three fusion proteins are folded similarly to their known or predicted structures of the individual proteins (Fig 6). For all the fusion proteins, the percentages of secondary structures predicted through molecular modeling and those obtained from CD spectra are in close agreement supporting the correctness of structures obtained through molecular modeling.

In antigen-antibody interactions, direct contact between the epitope and paratope of the antibody depends upon the hydophilicity and surface exposure of antigenic determinants. In addition to these, shape complemantarity, involvement of water molecules or other co-factors at the antigen-antibody interface may influence antigen-antibody interactions [50].

In all the fusion molecules, HSPX is the common component that contains one epitope which forms the beta sheet and coil as predicted through molecular modeling. In the case of HSPX-tnPstS1 and HSPX-PE35 fusion molecules, the epitope of HSPX protein retains their original conformation while in case of HSPX-fbpC1, whole of the epitopic region loses the native conformation and forms random coil (Fig 6A–6C). The data along with the altered configuration of HSPX may explain the decrease in the sensitivity of HSPX-FbpC1 (Fig 6F). Thus, Structure prediction of fusion molecules would be helpful for designing more efficient fusion antigens.

These antigen-antibody based assays could replace microscopy and culturing to diagnose TB in high burden TB endemic countries because of their speed and simplicity. Moreover, for extra-pulmonary cases, invasive procedures are used to get a sample for analysis. A serodiagnostic test based upon antibodies detection can replace such invasive procedures for getting samples from disease site. [3, 51–53]. WHO conducted the meta-analysis and systematic review of the accuracy of in-house ELISA kits for diagnosis of TB and developed the policy regarding the use of ELISA in 2011. Due to the inconsistent and variable ELISA results, WHO recommended that these tests should not be used for diagnosis of TB. However, they stated clearly in their 2011 policy that further research to identify new/alternative point-of-care tests for TB diagnosis and/or serological tests with improved accuracy is strongly encouraged. Appropriate study design including study population, eventual follow-up is important factors while performing such research [2]. Xpert^®^ MTB/RIF (Cepheid Inc.) I, a PCR-based assay, was recommended by WHO to diagnose MDR-TB and HIV-associated TB [2] but the high cost is a major barrier for wide scale application of this test in high TB burden countries afflicted with poverty. Therefore, efforts to enhance sensitivity of a relatively cheaper immune based assay need to be continued. Reliability and sensitivity of the serodiagnostic assay can be improved using and evaluating other antigens in this protocol.

## Conclusions

The fusion molecules HSPX-tnPstS1 and HSPX-PE35, reported for the first time, on account of their sensitivity and soluble expression seems to have high potential in developing a more reliable and cheaper serodiagnostic procedures for tuberculosis. Use of these fusion molecules in combination with other more efficient fusion molecules would be helpful in increasing the accuracy of the serodiagnostic assay and making the test more economical. Furthermore, the heat shock protein HSPX in fusion with other proteins seems to have a role in soluble expression of proteins in *E*. *coli*. As HSPX also contains T-cell epitopes, its fusion molecules seem to have the additional advantage as vaccine candidates.
